# The Prevention of Anxiety and Depression in Early Childhood

**DOI:** 10.3389/fpsyg.2020.517896

**Published:** 2020-09-30

**Authors:** Natalie Baughman, Susan L. Prescott, Rosanna Rooney

**Affiliations:** ^1^School of Psychology, Faculty of Health Sciences, Curtin University, Perth, WA, Australia; ^2^The ORIGINS Project, Telethon Kids Institute and the Division of Paediatrics, University of Western Australia, Nedlands, WA, Australia; ^3^School of Medicine, University of Western Australia, Nedlands, WA, Australia; ^4^Perth Children’s Hospital, Nedlands, WA, Australia

**Keywords:** early childhood, prevention programs, anxiety disorders, depressive disorders, mental health promotion, school-based programs

## Abstract

Recent statistics suggest that anxiety and depressive symptoms and disorders can occur earlier in life than previously thought, and appear to be on the increase. The burden that is associated with internalizing symptoms is large, with children’s social, emotional, and cognitive development negatively impacted. Research suggests that early intervention and prevention is vital for adaptive development, and this review set out to explore the literature regarding social-emotional learning programs for children of preschool age that aim to prevent and reduce symptoms of anxiety and depression. The review focused on interventions that could be delivered universally in the school context to children aged 4–6 years or their parents. Only six programs were identified that met these criteria. The results of this review suggest that intervention and prevention efforts in early childhood are needed and can be effective in terms of reducing the burden associated with internalizing symptoms in childhood, at least in the short term. This appears to be the case particularly when parents are actively involved in the intervention, too. However, more rigorous research is needed that involves larger randomized controlled trials with multiple reporters and consistent administration of assessments across the samples.

## Introduction

While universal school-based prevention programs offer promise in the context of anxiety and depression in late childhood and adolescence, few programs are currently available that are appropriate for children younger than 6 years of age. The lack of programs for young children is surprising, given that anxiety is the second most common disorder in childhood, affecting up to 9% of preschoolers worldwide (Skovgaard et al., 2005; Egger and Angold, 2006; Bufferd et al., 2012; Wichstrøm et al., 2013). Depressive disorders are less common in this age group (1.1%; Skovgaard et al., 2005; Egger and Angold, 2006; Bufferd et al., 2012; Wichstrøm et al., 2013); however, the impact of both anxiety and depression is extensive in terms of emotional, social, and economic costs, even in young children (WHO, 2014b; Lahey, 2015; AIHW, 2018; National Mental Health Commission, 2019). In fact, anxiety and depression are the second and third leading causes of the total disease burden for 5–14 years old children, surpassed in burden only by asthma (AIHW, 2019). In adolescence, suicide and self-inflicted injuries replace asthma as the leading cause, while anxiety and depressive disorders remain on ranks 2 and 3 (AIHW, 2019). These data indicate that both disorders are particularly troublesome across the age groups, and further suggest that their impact may be cumulative, with suicide and self-inflicted injuries ranking as the largest cause of the disease burden from age 15 onward. The continued large burden also suggests that current treatment efforts may not be effective enough in reducing the burden of anxiety and depression. The reasons for this are likely multiple, including the fact that only approximately 56% of children and adolescents with symptoms are receiving professional help (Lawrence et al., 2015).

Causal accounts for the development of anxiety and depressive disorders in early childhood are lacking, thus much of the research on the prevention of internalizing disorders focuses on symptoms and factors that appear to protect or place individuals at risk of developing internalizing disorders. These risks typically concern biological or behavioral factors in the child themselves, as well as to factors within their family, community, and wider environment (e.g., Côté et al., 2009; Bernaras et al., 2019). Child development occurs rapidly in the first year of life and is dependent upon a complex interplay between endogenous factors, such as temperament, social, emotional and cognitive competencies, and exogenous factors, such as parenting style and parental capacities. Difficulties in any one of these domains are likely to result in difficulties in the other. In early childhood, the largest risk for internalizing disorders relates to child temperament, such as behavioral inhibition or extreme shyness (Rapee et al., 2005), with some research evidencing a relationship between difficult temperament and anxiety and depressive symptoms from as young as 5 months of age (Côté et al., 2009). The second largest predictor for anxiety and depression in early childhood is maternal depression (Côté et al., 2009), suggesting that both genetic and behavioral factors are likely involved in child psychopathology. Other research points to the role of stress in the development of psychopathology (Grant et al., 2003). In early childhood, this includes for example, the experience of family dysfunction (Hammen et al., 2004), problematic attachment styles, poor or ineffective parenting practices, and low sense of maternal self-efficacy (Côté et al., 2009), all of which have been associated with the development of depression and anxiety in childhood (Lewis and Olsson, 2011; Bernaras et al., 2019).

Prevalence and burden of disease data and etiological models of anxiety and depression in combination with increasing research into the type, efficacy, and timing of treatments (e.g., Briggs-Gowan et al., 2000; Rapee, 2013) have prompted the development of programs aimed at preventing and reducing anxiety and depressive symptoms in our young people. Research suggests that the purposeful development of skills in the social, emotional, and cognitive domains is possible and can lead to improved outcomes in children and adolescents (see e.g., Weissberg et al., 2015). Mounting evidence suggests that prevention programs should commence at an early age (Bernaras et al., 2019).

Of particular interest are prevention programs that can be delivered universally to whole populations, regardless of symptom levels or risks.

A number of universal school-based programs have been offered in an attempt to prevent and reduce mental health symptoms in middle childhood and adolescence (see e.g., Rimm-Kaufman and Hulleman, 2015, for a review of social-emotional learning programs in primary schools). However, in light of the current data on the prevalence of mental health disorders in early childhood worldwide, it appears necessary to intervene much earlier than the middle school years (Bayer et al., 2011). While school-based prevention programs have been available for the upper primary and lower secondary years for some time, preschool programs are relatively new, and research concerning the prevention of internalizing disorders early in life is still in its infancy. A small number of prevention programs targeting internalizing symptoms specifically in preschool children does exist and is reviewed here. Most of the prevention efforts in preschoolers to date have focused on the reduction of externalizing, or behavioral problems in young children, rather than internalizing problems. This is likely due to the practical implications relating to classroom conditions that are caused by the more disorderly or disruptive behavior that is concomitant to externalizing difficulties. However, it is critical to support children with internalizing problems as soon as possible, so that the impact on their functioning and development can be minimized. Very few prevention programs currently exist for children of preschool age; however, the ones that do exist, and are reviewed here, deliberately target identified risk and protective factors.

This paper presents a review of social-emotional learning programs that are available for use with children aged 4–6 years and their parents. We will discuss the programs in terms of their theoretical underpinnings, aims, and evidenced outcomes, in order to establish what “works” and what does not “work” in the prevention of anxiety and depressive symptoms in young children.

## Materials and Methods

An initial search on the Australian website BeYou, a mental health promotion initiative for Australian schools, yielded three results for evidence-based programs deliverable in Australian schools in the early learning setting. After reviewing the programs, one program was removed, as it targets problem behaviors and school failure, leaving two programs to review. A second search in the BeYou programs directory with the search terms “parents,” “evidence,” “early learning,” “emotional and behavioral difficulties,” “families and parenting education,” “resilience,” “social and emotional learning,” “mentally healthy communities,” “family partnerships,” “learning resilience,” “early support,” and “responding together” yielded 20 results. After reviewing the programs, only two programs for parents remained that specifically targeted emotions.

Searches in the Cochrane Library using the search terms “anxiety,” “depression,” “prevention,” “early childhood,” and “preschool:” yielded 3,844 Cochrane reviews and 264,624 trials. After narrowing the search terms to include “child health” and “mental health,” 53 reviews remained. When narrowing the search further to include only trials and reviews involving children aged 4–5 years, three programs remained.

## Results

### Review of Programs Delivered to Children

#### Preschool PATHS Program

The Preschool Promoting Alternative Thinking Strategies (PATHS; Domitrovich et al., 2007) Program is a developmentally appropriate adaptation of the PATHS program for 3–6 year-old children. The PATHS program was originally designed as an intervention to develop emotion expression and reduce behavior problems in deaf children, but it is useful also for the prevention of internalizing symptoms and is widely used universally in mainstream classrooms. Theoretically, this program aligns with Affective-Behavioural-Cognitive-Dynamic (ABCD) model of development of Greenberg and Kusché (1993). The ABCD model posits that social-emotional competence results from the integration of emotional, behavioral, and thinking skills that include for example, impulse control (inhibition), identification of emotions, and problem-solving skills. A strong emphasis is placed on the role of parenting and attachment throughout development. Problems in any of these areas would result in difficulties in effective coping. The PATHS preschool curriculum consists of hundreds of lessons covering five core concepts: self-control, emotional understanding, building self-esteem, relationships, and interpersonal problem-solving skills. Activities are play-based and include modeling, stories, discussions, re-telling of stories, positive reinforcement techniques, and role plays. The self-control technique “Doing turtle” is explicitly taught early on, and involves specific hand actions and self-talk, designed to stop impulsivity. Classroom resources include story books, posters, hand puppets, and picture cards. Stickers are used to provide positive reinforcement, and information materials are also provided for parents (see Kusché and Greenberg, 1994, for a detailed description of the program and its materials). A number of randomized controlled trials (RCTs) have been conducted with the preschool version of the program (e.g., Domitrovich et al., 2007; Bierman et al., 2008; Arda and Ocak, 2012; Nix et al., 2013; Hughes and Cline, 2015). These studies have provided evidence for the efficacy of the program in terms of increased receptive and expressive emotion vocabulary [*F*(8, 166) = 8.86, *p* < 0.01; *ŋ*^2^ = 0.36 and *F*(8, 163) = 5.59, *p* < 0.05; *ŋ*^2^ = 0.37, respectively; Domitrovich et al., 2007; *β* = 0.25, *p* < 0.05; Nix et al., 2013], increased social competence [*F*(7, 187) = 17.62, *p* < 0.0001; *ŋ*^2^ = 0.48; Domitrovich et al., 2007] and competent social problem solving (*β* = 0.36, *p* < 0.01; Nix et al., 2013), and less social withdrawal [*F*(7, 187) = 4.44, *p* < 0.05; *ŋ*^2^ = 0.24; Domitrovich et al., 2007], compared to the control group children. Anxiety and depressive symptoms were not specifically assessed in these studies; however, Domitrovich et al. (2007) reported improvements in internalizing behavior in a rural subsample. Hughes and Cline (2015) reported increases in teacher-reported, but not in parent-reported strengths and difficulties questionnaire (SDQ; Goodman, 1997) at post-test for total strengths and difficulties [*F*(1, 47) = 7.03; *ŋ*^2^ = 0.23] and for all subscales, except the peer problems subscale. The impact on executive functioning was variable across the different studies, with some finding no reliable differences (e.g., Domitrovich et al., 2007) and others finding small effects in task orientation (*p* = 0.02; Bierman et al., 2008). In sum, these studies provide evidence that the Preschool PATHS Program is associated with positive change on emotional and social variables, as well as executive functioning in children of preschool age in the short term. These studies were robust and well-designed, with important confounds such as verbal ability, ethnicity and special needs status etc., accounted for in the analyses. Attrition was relatively low, with authors reporting between 3 and 18% at post-test for the various studies. Another strength was that executive functioning was assessed in addition to social and emotional skills. However, none of the studies included a follow-up of the samples beyond the post-test, which leaves unanswered the question of the program’s longer-term efficacy. Furthermore, while the use of multiple reporters, particularly the inclusion of observational data is undoubtedly strength in the design, teacher-reports may be unreliable due to potential bias toward the intervention, and possible fatigue relating to the number of children each teacher was completing measures for. Importantly, however, the data reported here support the notion that social-emotional skills may indeed be modified and improved in preschool aged children using the PATHS program. This means that this age range may present a window of opportunity for altering the trajectories that could lead to poor outcomes in the social-emotional domains.

#### Fun FRIENDS

The Fun FRIENDS (Barrett, 2007) program is a developmentally appropriate adaptation of the FRIENDS for Life program (Barrett and Ryan, 2004; Barrett, 2005) for preschool children. The Fun FRIENDS program aims to prevent anxiety and promote social and emotional skills and resilience in early childhood. The program utilizes developmentally appropriate adaptations of cognitive-behavioral therapy (CBT) principles, which posit that thoughts and feelings are closely connected. The CBT approach is based on the assumption that psychopathology is the result of dysfunctional assumptions about the self and the world; that is, negative thoughts generate and perpetuate negative emotional states. Therapy under this approach aims for recognition and subsequent restructuring of negative or dysfunctional thinking patterns.

The Fun FRIENDS program involves parents, teachers, and students. Parents attend a number of information sessions, and are encouraged to complete an accompanying workbook at home. Teachers attend compulsory training workshops prior to program implementation. The school-based program consists of 9 weekly lessons, which are 1–1.5 h in duration. Program content is delivered in experiential play-based activities and aims to develop cognitive-behavioral skills, including recognizing feelings and body clues in the self and in others, identifying and practicing positive coping strategies and behaviors, identifying helpful and unhelpful self-talk, and then challenging and changing unhelpful thoughts. Children are also taught how to develop a graded exposure hierarchy with help of their parents and teachers. Emotion regulation and positive social skills are also included in this program. It is recommended that the program is delivered to several smaller groups of 4–5 children, who form a large group at the end of the session for a general discussion.

Several studies have been conducted with the Fun FRIENDS program, and two are reviewed here. The authors report reliable pre‐ to post-test gains on teacher-reported emotional and behavioral strengths [*F*(1,261) = 8.63, *p* < 0.005, partial *η*^2^ = 0.03; Pahl and Barrett, 2010], with the largest increase in scores observed in the girls in the intervention group [*F*(1,259) = 9.81, *p* < 0.005] and at 12-month follow-up (Anticich et al., 2013), as well as reliable improvements in anxiety symptoms in the intervention group from pre-test to 12-month follow-up (*p* < 0.05), but not at post-test (Pahl and Barrett, 2010). In the second study, reliable pre‐ to post‐ to follow-up test differences were reported for emotional and behavioral strengths, behavioral difficulties, and behavioral inhibition, although effect sizes were not reported. It is important to note, however that both studies presented with significant challenges relating to their research design, samples, and data. For example, problems with the sampling methods were noted whereby the participating schools in the first study, while randomly allocated to either an intervention or control group, initially self-selected participation in the study at a conference. The authors additionally noted a lack of adherence and social validity, although exact details were not provided. Furthermore, a large proportion of post-intervention data was unavailable for analysis both at post-test and at follow-up, with almost half of the sample affected in both studies. Attrition at post-test was reported between 41 and 43% for parent report and less than 2% for teacher report in the 2010 study. The authors explain that this was due to ethical constraints, but effectively, this means that the results of both studies are difficult to interpret, particularly given that in the first study, 12-month follow-up data were only collected for the intervention group, so that comparisons to a control group were impossible. The second study was conducted entirely in Catholic schools, which may limit the generalizability of the results to the general population. Effect sizes are also very small. More research is needed to fully evaluate the efficacy of this program.

#### “Play Skills for Shy Children” or “Social Skills Facilitated Play” (SST)

Play Skills for Shy Children (Coplan et al., 2010) is a targeted intervention program for extremely inhibited preschoolers that aims to prevent and reduce social anxiety. The authors propose that extreme shyness or behavioral inhibition in childhood is a major risk factor for later anxiety disorders and social anxiety disorder in particular (Coplan et al., 2010). The SST program was based on the assumption that the development of social skills in early childhood would lessen the risk of internalizing problems. The program was further developed as an alternative to CBT-based programs, as the cognitive demands that CBT programs command were assumed too advanced for young children. The SST program consists of seven weekly 1-h sessions and one booster session 4 weeks later. Sessions involve a combination of free and structured play, and new didactic content focusing on specific social skills is delivered in each session. The sessions are delivered by trained staff in small group settings, outside of school. The program is based on social skills training paradigms and includes modeling, training, coaching, emotion-regulation and relaxation, and a peer play component that aims to promote social interaction beyond the training program. The content of the program focuses on developing skills such as initiating and maintaining peer interactions and identifying emotions in the self and others. The program also aims to develop children’s emotion expression and affect regulation, coping skills, and social problem solving and relaxation. Sessions include circle time and free-play with the didactic content delivered using songs, games, puppets, and discussions. The children practice the new skills in free play sessions that are leader-guided and facilitated to promote social interactions.

The program has shown preliminary efficacy in a small targeted sample in terms of reductions at post-test in reticent-weary behaviors (*p* < 0.05, *ŋ*^2^ = 0.182) and increases in social competence (*p* < 0.05, *ŋ*^2^ = 0.202), but no effects on teacher-rated anxious behaviors and prosocial behaviors (Coplan et al., 2010). This was a very small (*n* = 22) wait-list control trial conducted over two consecutive years. The intervention was delivered outside of school with child assessments occurring at preschool. The results provide some preliminary support for the efficacy of the SST program for highly inhibited children of preschool age, at least in the short-term on behavioral variables, but not for anxiety symptoms. Strengths of this study include the use of observational measures and the fact that observers and teachers were blind to the study conditions. A number of limitations are noted, including the very small sample (*n* = 22 at post-test) and lack of follow-up beyond the post-test. While observational data collected by blind assessors are advantageous in terms of reducing biases in favor of a particular group, it is still associated with important shortcomings, such as inter-rater disagreement, and the reliance on subjective interpretations of the reviewers. On a practical note, while targeted application is useful for examining the efficacy of a prevention program, universal application of this particular program would be rather expensive and would not be feasible on a larger scale, or longer-term. Furthermore, targeted implementation limits the children’s exposure to the modeling of and interaction with more competent peers and may limit generalizability of skills. Compliance may be an issue, depending on the location and accessibility of the sessions, and stigma is likely a concern, due to the selective nature of program inclusion.

### Review of Programs Delivered to Parents

Parents are a child’s first educators, and research shows the enormous impact parents and carers have on the development of their children. Given the importance of parenting on child outcomes, interventions have been developed that aim to enhance parenting skills, and provide education on child development, mental health disorders, and principles of parenting and attachment.

#### Cool Little Kids

“Cool Little Kids” (Rapee et al., 2005) is a targeted prevention and early intervention program for parents with children aged between 3 and 6 years; its aim is to prevent the development of anxiety in preschool children. The Cool Little Kids program is developed on the basis of models that give central importance to withdrawn behavior in the development of anxiety (e.g., Rapee, 2001), as well as research into risk factors, such as overprotective parenting, for example. Cool Little Kids is a psycho-educational program for parents, which also teaches cognitive-behavioral strategies, such as exposure hierarchies and cognitive restructuring. It is conducted as a group program with six 90-min sessions – 4 weekly sessions plus one session after 2 weeks and the last session 4 weeks later. In addition to providing information on anxiety in childhood, the program covers parent management techniques, including the effect of overprotection on anxiety and discussions of potential high-risk periods, such as starting school. Parents learn to develop graded fear exposure strategies with their children and learn how to support their child in completing the steps.

One RCT was conducted with a targeted sample consisting of parents with 146 preschoolers who displayed severe behavioral inhibition, most of whom also had an anxiety disorder (Rapee et al., 2005). The intervention was associated with a significant reduction in the number of anxiety disorders in the children after 12 months [*χ*^2^ (1, *N* = 146) = 0.34, *p* = 0.53]. The number of sessions attended also predicted anxiety disorders at 12 months [*χ*^2^ (1, *N* = 146) = 3.35, *p* = 0.07] behavioral inhibition however did not change. The authors report attrition of 20.5% at the 12-month post-test. An additional long-term follow-up assessment was conducted 11 years later in a smaller sub-sample (Rapee, 2013). Analyses revealed that the girls in the intervention group showed significantly fewer internalizing disorders [*F*(1,58) = 4.42, *p* = 0.040, *μ*_p_^2^ = 0.071], anxiety symptoms [*F*(1,58) = 9.12, *p* = 0.004, *μ*_p_^2^ = 0.136], and self-reported life interference from anxiety [*F*(1,58) = 5.38, *p* = 0.024, *μ*_p_^2^ = 0.085] at the 11-year follow-up, compared to the girls in the control group. These differences were not apparent for the boys in this sample. An online version of this program was also trialed and yielded significant reductions in child anxiety symptoms. These studies support the notion that even brief parenting programs can improve child anxiety outcomes at 12 months. It is remarkable that a group difference was evident after 11 years, and it would be useful to conduct further studies into the long-term effectiveness of this training program for parents.

#### Tuning Into Kids

Tuning Into Kids (TIK; Havighurst and Harley, 2007) is a program for parents of children aged 4–5 years that aims to help parents enhance their children’s behavior and emotion knowledge. The program is based on theories of emotion socialization and attachment and utilizes the concept of emotion coaching (Cassidy, 1994). TIK is based on the premise that improvements in parents’ emotional competence and ability to coach their offspring’s emotions, and improves the child’s emotional knowledge and behaviors. The intervention consists of 6 weekly group session, each 2 h in duration that cover psycho-education and emotion coaching principles. Teaching methods include discussions, role plays, demonstrations, DVD materials, and information sheets. Parents are first taught about emotion awareness and regulation in relation to their own emotions, including a range of relaxation, problem-solving, and self-regulation strategies before learning to apply these to their parenting approach. Two efficacy trials have been conducted [Havighurst et al., 2009 (study 1); Havighurst et al., 2010 (study 2)] with around 200 parents each. In the first study, the intervention was associated with significant increases in parents’ self-reported emotion coaching [*F*(1,181) = 31.47, *p* < 0.001, partial *η*^2^ = 0.15] and emotion dismissing [*F*(1,181) = 58.67, *p* < 0.001, partial *η*^2^ = 0.25]. Reliable differences were also noted in relation to child behavior intensity [*F*(1,181) = 18.39, *p* < 0.001, partial *η*^2^ = 0.09] and “clinically relevant child behavior” reduced by 23% in the intervention, compared to an increase of 2% in the control group; however, statistical information, including effect sizes is not provided. In the second study, post-testing revealed reliable differences on parents’ self-reported emotion awareness and regulation (*d* = 0.29), emotion dismissing (*d* = 0.86), emotion coaching (*d* = 0.64), and empathy/connection (*d* = 1.08). Differences were also apparent for emotion labels (*d* = 0.57) and emotion exploration (*d* = 0.66), which were derived from video observations, favoring the intervention group (Havighurst et al., 2010; *n* = 216). In this study, reliable differences are reported for all children regardless of study conditions, with the children in the intervention group showing reliably better emotion knowledge than the control group children: child emotion knowledge (*d* = 1.00 for the intervention group and *d* = 0.52 for the control group). The authors note attrition of 16.2% at post-test and 12% at 6-month follow-up.

In conclusion, these studies found that the TIK program is associated with increased self-reported parent emotion competence, increased child emotion knowledge (vocabulary), and reduced child problem behavior in the short to medium term. The studies contribute important evidence to the literature on the role of parenting practices in child behavior outcomes. However, limitations were apparent on both studies; specifically, in the first study, only parent-reported data were included, which is prone to expectation biases and only pre‐ and post-tests were conducted, without any longer term follow-ups. In study 2, multi modal reporting and a 6-month follow-up were included, which both strengthen the validity of the results. It would have been useful to also include other measures to validate the authors’ arguments relating to the importance of improved emotion knowledge to empathy and social skills, particularly close relationships, for both, the parents and the children in this study. More research is needed to validate the efficacy of this program.

#### Triple P: Positive Parenting Program

The Triple P – Positive Parenting Program (Sanders, 1999) is a psycho-education program for parents, which aims to develop parent knowledge, skills, and confidence in relation to parenting pre-adolescent children. The overarching aim of the Triple-P program is to prevent severe emotional and behavioral problems in children of all ages. The program is based on social learning theory (e.g., Patterson et al., 1982), and utilizes behavior change principles (e.g., Risley et al., 1976) to develop parenting practices. Parents are taught to self-regulate emotions and behaviors in themselves, so that they are better able to manage child behavior, and help their children learn how to self-regulate (Sanders, 1999). A number of distinct programs (modalities) are available to parents with children of different ages (infancy to adolescence) and needs (severe behavior problems, disabilities, divorce, family dysfunction, overweight children, indigenous families, and anxiety). Delivery of the program content is flexible and can occur in groups, one-to-one, on the telephone, or as a self-directed program. The Triple-P program was designed as a multi-level population health intervention with utility as a prevention or intervention program that can be delivered by a range of health or community professionals. The standard Triple-P program for parents of young children consists of sessions that aim to teach parents 17 child management strategies that are designed to promote child competence and development, and help parents manage difficult child behavior. A multitude of studies have been conducted worldwide that demonstrate the program’s overall effectiveness at improving wellbeing, parenting skills, family relationships, child mental health, and behavior (see e.g., Nowak and Heinrichs, 2008). A smaller number of studies has been conducted with parents of preschoolers specifically, with most studies however addressing behavioral, rather than emotional difficulties. Many of these studies were conducted with families from low socio-economic backgrounds. One study (Bor et al., 2002) was conducted in a disadvantaged, low SES sample, in which the families were selected on the basis of the children’s levels of problem behaviors. This study compared a standard PPP version to an enhanced version and a control group, and found reliable improvements on all measures of child behavior, and mother-reported parenting skills, confidence, satisfaction, and competence at post-test for both intervention groups. At the 12-month follow-up, a main effect for decreases in inattentive behavior was found for the children whose parents had completed an enhanced version of the program. Another trial evaluated a self-directed version of the Triple-P program among parents of preschool-age children at risk of developing conduct problems (Markie-Dadds and Sanders, 2006) who were already showing elevated behavior difficulties. The parents received three booklets designed to help them acquire new parenting skills. Analyses revealed reliable group differences at post-test on measures of child behavior, parenting style, and parents’ sense of competence favoring the intervention group, but no differences at 6-month follow-up. Together, these results provide evidence for the efficacy of the Triple-P parenting program in improving parenting competence and reducing child behavior problems in young children at risk of behavioral difficulties or with early onset disruptive behavior and attentional/hyperactivity difficulties. Several limitations of the studies are evident, namely, the lack of standardized clinical measures assessing disruptive behavior symptoms in study 1, and use of parent report measures in both studies. Self-report measures assessing parenting practices are most probably prone to biases. Furthermore, the wait-list control groups were not assessed at the follow-ups, which does not allow for inferences to be made beyond the post-test.

## Conclusion and Future Directions

The programs reviewed and presented here suggest that intervention and prevention efforts in early childhood are needed and can be effective in terms of reducing the burden associated with internalizing symptoms in childhood, at least in the short term. Comprehensive skills training in social, emotional, and cognitive domains appear most useful, in programs targeted at children or parents. The programs reviewed here have demonstrated efficacy relating to a number of outcomes, but most if not all studies have had moderate to severe methodological limitations that impact on the validity of the results and the generalizability of the improvements described. Most noteworthy is the consistent lack of follow-up beyond the post-test. Short-term gains are very promising and important findings, but follow-ups beyond 6 and 12 months are needed, to evaluate the long-term efficacy of the interventions. Research using universal prevention programs in older age groups have shown that prevention effects are often not observable immediately after the intervention, but in fact manifest at later times, probably because skill development may take some time (e.g., Misfud and Rapee, 2005; Rapee et al., 2005; Gilham et al., 2006). It remains to be seen whether this is the case also for children of younger ages. Other problems within this literature revolve around the types of assessment measures chosen, as well as the frequency and consistency of testing. For example, it is difficult to accurately assess anxiety and depressive symptoms in early childhood. This is due to a number of reasons. For example, there are relatively low prevalence rates of internalizing disorders in this age group, and disorder categorizations are poorly-defined for very young children (e.g., Tandon et al., 2009). There are also difficulties related to the use of self-report measures with very young children, given the developmental limitations in language and cognitive ability of young children. There is also a vast literature describing discrepancies between parent-report and child self-report on measures assessing internalizing symptoms. In older children and adolescents, more weight is given to the direct child measures, but in younger children this may not be possible, given the constraints just discussed. As an alternative, many studies have included videotaped observations that are subsequently evaluated by multiple raters; however, these recordings typically provide only a snapshot of behavior, which may not necessarily be representative of overall behavior. Furthermore, measures completed by persons not blinded to the condition, such as parents and/or teachers are also prone to biases and errors. Several of the studies reviewed here also unfortunately administered the assessments inconsistently to sub-samples, rather than the entire sample, or followed-up only a sub-sample, rather than the whole sample of children. Surprisingly, few studies utilized tests of executive functions or cognitive processes, even though in the literature a clear argument exists for a relationship between cognition, emotions, and behavior, and given that most of the programs reviewed here utilized cognitive theories and approaches. More recent research also points to a connection between physiological markers, such as cortisol, inflammatory markers, and the gut microbiota and anxiety and depression, which was not investigated in the studies reviewed here. Given the difficulties associated with assessing mental health symptoms and stress in young children, more recent studies have included evaluations of physiological markers. For instance, in their RCT on the effectiveness of the Tools of the Mind program in a preschool sample, Blair and Raver (2014) incorporated an evaluation of levels of salivary cortisol and alpha amylase in their sample. Data from adult samples are also available that connect levels of inflammatory markers obtained from saliva samples with affect in healthy samples (Stellar et al., 2015). Other research has found associations between the gut microbiome and mental health, providing evidence for the gut-brain axis and the involvement of the gut in mental health outcomes. The inclusion of physiological stress markers (e.g., saliva, stool, or gut samples of the children) has the potential to reduce reporting bias associated with verbal and observational reports, and may circumvent the problem of disagreement between parent‐ and child-reported data. Furthermore, physiological assessments, as well as tests of cognitive functioning, offer objectivity that current and traditional assessments, such as questionnaires and behavioral observations simply do not.

We conclude that while the programs reviewed here provide promising support for the effectiveness of prevention programs in early childhood, more rigorous research studies are needed that involve longer-term RCTs, which take into account multiple reporters, and that administer assessments of social, emotional, cognitive, and physiological variables equally across the sample, with all confounds controlled for.

We propose to develop a new program for preschool-aged children and their parents/carers that utilizes the findings discussed in this review. This new program is a universal program that can be implemented in kindergarten and preschool settings by trained teachers, with an associated parent component. The program includes skill development for children, parents/carers, and teachers, as well as important psycho-education for parents and teachers relating to symptoms, management, and referral of mental health difficulties. A longitudinal RCT within the local cohort study called ORIGINS will be run to evaluate the efficacy of this program. Assessments will occur within social, emotional, cognitive, and physiological domains controlling for SES, and child verbal ability. Parent, teacher, and child-assessments will be utilized, as well as behavioral observations conducted by blinded assessors. Additionally, a longitudinal randomized controlled study design over at least 3 years would allow for the identification and tracking of early markers in the development of anxiety and depression in young children. This would also help address questions in the literature relating to longitudinal continuity of childhood anxiety and depression (e.g., Luby et al., 2009).

## Author Contributions

NB was the lead author of the review. She researched and wrote content for the article. All authors contributed to the article and approved the submitted version.

### Conflict of Interest

The authors declare that the research was conducted in the absence of any commercial or financial relationships that could be construed as a potential conflict of interest.
